# Regulatory T Cells and Human Disease

**DOI:** 10.1155/2007/89195

**Published:** 2008-01-01

**Authors:** Nathalie Cools, Peter Ponsaerts, Viggo F. I. Van Tendeloo, Zwi N. Berneman

**Affiliations:** ^1^Vaccine and Infectiouse Disease Institute (VIDI), Laboratory of Experimental Hematology, University of Antwerp, 2610 Antwerp, Belgium; ^2^Center for Cellular Therapy and Regenerative Medicine, Antwerp University Hospital, 2650 Antwerp, Belgium

## Abstract

The main function of our immune system is to protect us from invading pathogens and microorganisms by destroying infected cells, while minimizing collateral damage to tissues. In order to maintain this balance between immunity and tolerance, current understanding of the immune system attributes a major role to regulatory T cells (Tregs) in controlling both immunity and tolerance. Various subsets of Tregs have been identified based on their expression of cell surface markers, production of cytokines, and mechanisms of action. In brief, naturally occurring thymic-derived $\text{CD}4^{+}\text{CD}25^{+}$ Tregs are characterized by constitutive expression of the transcription factor FOXP3, while antigen-induced or adaptive Tregs are mainly identified by expression of immunosuppressive cytokines (interleukin-10 (IL-10) and/or transforming growth factor-β (TGF-β)). While Tregs in normal conditions regulate ongoing immune responses and prevent autoimmunity, imbalanced function or number of these Tregs, either enhanced or decreased, might lead, respectively, to decreased immunity (e.g., with tumor development or infections) or autoimmunity (e.g., multiple sclerosis). This review will discuss recent research towards a better understanding of the biology of Tregs, their interaction with other immune effector cells, such as dendritic cells, and possible interventions in human disease.

## 1. INTRODUCTION

The main function of our immune system is to protect us from invading pathogens and microorganisms by destroying infected cells, while minimizing collateral damage to tissues. Within the large pool of different immune effector cells, the recently rediscovered regulatory T cells (Tregs) play an important role in controlling immune responses and silencing self-reactive T cells.

## 2. ORIGIN AND SUBSETS OF REGULATORY T CELLS

Tregs were described for the first time in the early 1970s and were called suppressor cells [1, 2]. Despite many efforts, this research topic was abandoned in the late 1980s due to difficulties in correctly identifying and isolating the suppressor cells. In 1995, Sakaguchi et al. [3] showed that the interleukin-2 receptor *α*-chain (CD25) could serve as a phenotypic marker for CD4^+^ Tregs. These observations led to the revival of Tregs, and this research field has evolved rapidly ever since. Currently, various subsets of both CD25^+^ and CD25^−^ Tregs populations have been described [4] (see Table 1). Different Tregs subsets are now subdivided based on expression of cell surface markers, production of cytokines, and mechanisms of action.

Naturally occurring thymic-derived CD4^+^CD25^+^ Tregs are a T cell population with immunosuppressive properties that constitutes 5–10% of the total peripheral CD4^+^ T cells [5, 6]. Besides the expression of CD25, they constitutively express other several activation markers, such as the glucocorticoid-induced tumor-necrosis factor (TNF) receptor-related protein (GITR), OX40 (CD134), L-selectin (CD62 ligand (CD62L)), and cytotoxic T lymphocyte-associated antigen 4 (CTLA-4 or CD152). However, it should be noted that none of these markers exclusively identifies Tregs as they can also be expressed to various degrees on activated T cell subsets and various antigen-presenting cells (APCs). More recent studies have identified the transcription factor forkhead box P3 (FOXP3) as a more exclusive intracellular marker for the identification of Tregs [7, 8]. In addition, FOXP3 is also a crucial transcription factor for the development and functionality of CD4^+^CD25^+^ Tregs. Loss of function mutations in FOXP3, both in mice and men, results in the absence of Tregs, leading to a phenotype with severe autoimmune disorders [9, 10], known as scurfy mice and IPEX (immunedysregulation, polyendocrinopathy, enteropathy, X-linked syndrome) in men. The important function of FOXP3 was also confirmed by studies showing that ectopic expression of FOXP3 in T cells leads to the generation of cells with a regulatory phenotype and a suppressive function [7, 11]. In addition, with regard to the biological function of FOXP3 in Tregs, it was demonstrated that FOXP3 blocks the ability of the Rel-family transcription factors NFAT and NF*κ*B to induce their target genes [12–14], and as a consequence, it acts as a transcriptional repressor of IL-2 and other cytokine genes (IL-4 and IFN-*γ*), thereby programming a cell not to exert immune stimulatory functions. Moreover, FOXP3 expression has also been demonstrated in activated T cells in humans [15], presumably acting as a negative feedback in order to control ongoing immune responses.

There is still ongoing discussion whether CD4^+^CD25^+^ Tregs originate in the thymus and constitute a separate lineage or they are generated from mature T cells in the periphery. Most likely, both origins seem to play an important role. During early life, Hassall’s corpuscules, epithelial substructures in the thymus, play an important role in the generation of Tregs [16, 17]. In addition, the expression of FOXP3 as a Treg lineage specification factor [18] also supports the notion that Tregs are a separately derived T cell lineage. Moreover, neonatal mice that have undergone thymectomy spontaneously develop autoimmune diseases [19, 20]. On the other hand, while thymic function is largely reduced after puberty in man, Tregs persist throughout life. This implies that (all) Tregs might originate from a pool of self-renewable long-term surviving thymic emigrants. However, Akbar et al. [21] recently showed that the number and function of CD4^+^CD25^+^FOXP3^+^ Tregs are maintained in humans even after the age of 70 years. Therefore, they suggested that these cells most probably do not derive from the thymic lineage of Tregs, but they are generated from the peripheral pool of CD4^+^CD45RO^+^CD25^−^FOXP3^−^ memory T cells.

Furthermore, several other studies also reported the existence of various subsets of antigen-induced or adaptive Tregs. The suppressive function of these induced Tregs is mediated by the production of suppressive cytokines (IL-10 and transforming growth factor-*β* (TGF-*β*)). Therefore, the current classification of induced Tregs is based on expression of different suppressive cytokines. CD4^+^ regulatory T cells of type 1 (Tr1) express high levels of IL-10 and moderate levels of IL-5, IFN-*γ*, and TGF-*β*, and they are negative for IL-2 and IL-4 [22, 23]. T helper 3 (Th3) regulatory T cells express high levels of TGF-*β* [24, 25]. Both types of induced Tregs equally suppress Th1^−^ as well as Th2^−^ mediated immune responses. Tr1 and Th3 have been shown to originate from naive resting T cells after stimulation with dendritic cells (DCs) [26], depending on DC type and activation status. In addition, naturally occurring Tregs are also involved in the generation of induced Tregs, a mechanism proposed as “infectious tolerance.” The latter is based on expression of certain integrins by naturally occurring CD4^+^CD25^+^FOXP3^+^ Tregs [27]. While *α*_4_ *β*_7_ integrin expression induces IL-10 producing Tr1 cells, *α*_4_ *β*_1_ integrin expression induces TGF-*β* producing Th3 cells. Furthermore, we have recently described an additional population of TGF-*β* and IL-10 double-positive CD4^+^CD25^−^FOXP3^−^ adaptive Tregs [28], induced after in vitro culture of peripheral blood lymphocytes (PBLs) with immature and mature DCs. Moreover, the suppressive capacity of this CD4^+^ T cell population was transferable to already activated antigen-specific CD8^+^ T cells when CD4^+^ T cells were conditioned by immature DCs, but not when CD4^+^ T cells were conditioned by Toll-like receptor-3 (TLR3) ligand-matured DCs.

Next to the involvement of CD4^+^ naturally occurring and induced Tregs in controlling proper function of the immune system, CD8^+^ T suppressor cells have also been described. CD8^+^ T suppressor cells are derived from an oligoclonal T cell population, and they lack CD28 and express FOXP3, GITR, CTLA-4, OX-40, and CD62L at the same level as compared to CD4^+^CD25^+^ Tregs [29, 30]. In addition, CD8^+^ T suppressor cells, that are able to inhibit T cell proliferation, can be induced by xenogenic APCs or by peptide-pulsed autologous APCs [31, 32].

Of note, a special population of natural killer (NK) cells and NKT cells with regulatory function has also been described. Their immune suppressive function is mediated by secretion of various cytokines (IL-13, IL-4, IL-10) or by direct cell-cell contact [33]. In this review, however, we will focus on the different subsets of Tregs.

## 3. MECHANISMS OF SUPPRESSION

All Tregs, both naturally occurring and induced, need T cell receptor (TCR) triggering for their suppressive function. However, once activated, their suppressive activity seems to be antigen-nonspecific [34]. To date, the precise mechanism(s) by which Tregs suppress effector T cell activation and/or function remains unclear. Moreover, results from many in vitro and in vivo studies or studies performed on mice and men are sometimes contradictory.

### 3.1. Cell-cell contact

**Figure 1:**
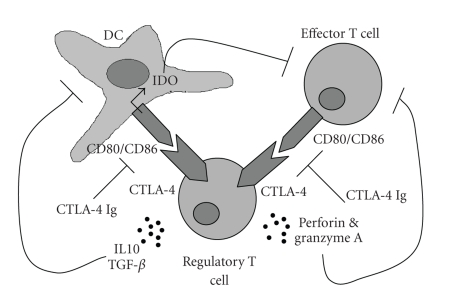
Possible mechanisms of suppression by regulatory T cells (Tregs). Tregs mediate their suppressive action by direct cell-cell contact mediated by CTLA-4 on both effector T cells as well as antigen-presenting cells (APCs), such as dendritic cells (DCs). Production of immunosuppressive cytokines, such as IL-10 and TGF-*β*, suppresses DC maturation, making DCs tolerogenic. Moreover, Tregs can kill effector T cells by expression of perforin and granzyme A. The figure also indicates therapeutic action of the anti-CTLA-4 antibody.

Several in vitro studies have demonstrated that CD4^+^CD25^+^ Tregs suppress proliferation and IFN-*γ* production by effector T cells through a direct cell-cell contact-dependent stimulation between suppressor and effector cells, possibly mediated by the expression of their cell surface markers GITR and CTLA-4 [35]. Ligation of CD80/CD86 on effector cells may transmit suppressive signals after engagement by cell surface CTLA-4 on suppressor cells, and it results in inhibition of effector T cell function (see Figure 1) [36]. Another mechanism for Tregs to affect effector T cell activation can be established by modulating DC function. Ligation of CD80/CD86 on DCs by CTLA-4 on suppressor cells results in expression and activation of indoleamine 2,3-dioxygenase (IDO) [37], a catabolic enzyme involved in tryptophan degradation. Reduced tryptophan concentration in culture medium has been reported to be associated with decreased activation of T cells and T cell deletion [38, 39]. Also, in several in vivo models for disease disorders, it was demonstrated that CTLA-4 blockade abrogates the suppressive function of murine (e.g., inflammatory bowel disease [40]) and human (e.g., melanoma patients [41–44]) Tregs. These results indicate that CTLA-4 plays a functionally significant role in Treg suppressive activity. On the other hand, CTLA-4 knockout mice appear to have cells that express the Treg-specific transcription factor FOXP3 and that are capable of suppression [45, 46]. These observations reveal that CTLA-4 is not the only accessory molecule required for Treg function.

Indeed, cell surface-bound TGF-*β* has been reported to mediate cell-cell contact-dependent immune suppression by CD4^+^CD25^+^ Tregs [47]. However, the latter remains controversial as functionally suppressive CD4^+^CD25^+^ Tregs can be isolated from TGF-*β* deficient mice [34]. In addition, CD4^+^CD25^−^ T cells transduced to express a dominant negative TGF-*β* receptor are still susceptible for Treg suppressive activity. Moreover, inhibition of T cell proliferation in vitro by IL-10 secreting Tr1 cells has been demonstrated to be independent of IL-10 production [48, 49]. Also O’Garra and Vieira [50] postulated that the regulatory activity of IL-10 secreting Tregs might be in competition with effector T cells for APC contact or for survival factors (e.g., IL-2). Therefore, contact-dependent suppression mechanisms might be dominant in vitro, circumventing the requirements for long-range suppressive cytokines.

### 3.2. Soluble factors

While above described results suggest a contact-dependent cytokine-independent mechanism of T cell suppression by Tregs, other in vitro studies clearly demonstrate that Tr1 cells and Th3 cells mediate their suppressive activity by producing immunosuppressive cytokines, IL-10, and TGF-*β*, respectively [51, 52]. Therefore, a definitive explanation regarding the in vitro suppressive mechanism of Tregs remains unclear due to well-known limitations of in vitro cellular assays differing in different laboratories. However, several in vivo studies have indicated the role of immune suppressive cytokines in Treg-mediated activity. Their involvement might be affected by many physiological factors, including the nature of the target organ and the magnitude of inflammation. Indeed, some autoimmune diseases are caused by IL-10 deficiency (e.g., colitis) [53, 54], whereas other autoimmune diseases are IL-10-independent (e.g., gastritis) [55] and/or -dependent on TGF-*β* deficiency (e.g., diabetes) [56]. Furthermore, CD4^+^CD25^+^ Tregs can be activated to express granzyme A and kill activated CD4^+^ and CD8^+^ T cells through a perforin-dependent mechanism, while Fas ligation has been demonstrated not to be involved [57, 58]. In addition, Tregs prevent DC maturation and activation through secretion of cytokines, both in mice and men. For this, IL-10 impairs the antigen-presenting capacity by downregulating MHC class II and costimulatory molecules on DCs [59]. TGF-*β* also downregulates MHC class II expression and prevents upregulation of costimulatory molecules [60, 61]. In addition, CD8^+^ suppressor cells, from healthy volunteers as well as transplant patients, have also been shown to inhibit upregulation of costimulatory molecules (CD80/CD86) on DCs and, importantly, increase the expression of Ig-like transcripts 3 (ILT3) and ILT4 on DCs [62]. These ILT molecules belong to the family of Ig-like inhibitory receptors and they are functionally related to killer cell inhibitory receptors. Ligation of ILT in antigen-presenting cells inhibits Ca^2+^ mobilization and tyrosine phosphorylation [63–65]. Moreover, such ILT-expressing DCs were shown to convert CD4^+^ allo-reactive T cells towards Tregs with immune suppressive function [66].

## 4. REGULATORY T CELLS IN HUMAN DISEASE

### 4.1. Autoimmunity

Reduced functional activity of Tregs results in an increased susceptibility to autoimmune disease. Patients with multiple sclerosis (MS) [67], polyglandular syndrome of type II [68], active rheumatoid arthritis (RA) [69], type-I diabetes [70], psoriasis [71], and myasthenia graves [72] show a significant decrease in the suppressive function of CD4^+^CD25^+^ Tregs as compared with cells from healthy donors. Because the percentage of CD4^+^CD25^+^ Tregs in peripheral blood of these patients is unaltered as compared with healthy controls, it has been suggested that it is mainly defective Treg function, rather than its number, that contributes to disease development in these disease conditions. In addition, in some autoimmune diseases, reduced levels of CD4^+^CD25^+^ Tregs have been observed in the peripheral blood of patients [73, 74]. However, in these cases, the recruitment or migration of Tregs from the blood to the inflammatory site may be responsible for the decreased number of Tregs in peripheral blood. Indeed, studies on patients with RA or juvenile idiopathic arthritis (JIA) demonstrated that at the site of inflammation (i.e., in the synovial fluid) the percentage of CD4^+^CD25^+^ Tregs was significantly increased as compared with the percentage in peripheral blood [75].

In addition to the autoimmune diseases described above, in allergic patients there is strong evidence for a dysfunction of CD4^+^CD25^+^ Tregs in suppressing Th2 responses [76, 77]. In individuals with allergic or asthmatic disease, a decrease and/or dysfunction of IL-10 secreting Tr1 cells was observed as compared to healthy individuals [78–81].

### 4.2. Cancer

Although the physiological function of Tregs is central for maintaining self-tolerance, this negative regulatory activity can also be counterproductive as Tregs might also suppress bonafide immune responses against tumors and viral infections. High numbers of CD4^+^CD25^+^ Tregs have been found in lung, pancreas , breast, liver, and skin cancer patients, either in the peripheral blood or around and within the tumor [82–86]. Moreover, Tregs isolated from tumors of lung cancer patients demonstrated potent immune suppressive activity of autologous peripheral blood T cells stimulated by anti-CD3 or anti-CD3/anti-CD28 in vitro [87]. Therefore, it can be postulated that Tregs can impair antitumor immune responses in cancer patients. In addition to naturally occurring CD4^+^CD25^+^ Tregs, also IL-10 producing Tr1 cells have been demonstrated to contribute to ineffective antitumor immune responses in cancer patients [88, 89]. In human ovarian tumors, it is demonstrated that plasmacytoid DCs induce IL-10 secreting CD8^+^ regulatory T cells capable of suppressing antitumor immunity through IL-10 [90]. In addition, Curiel et al. [91] described that tumor cells and surrounding macrophages produce the CCL22 chemokine, which mediates Treg-trafficking to the tumor through CCR4, thereby possibly contributing to the immune privileged features of these tumors. This observation was recently also confirmed in B cell non-Hodgkin lymphomas [92]. Furthermore, it is now believed that increased frequencies of Tregs in cancer patients are associated with a high mortality and reduced disease-free survival [93–95].

### 4.3. Infectious diseases

Several studies have also reported involvement of Tregs in infectious diseases, as Tregs might affect the magnitude of the immune response and therefore the outcome of viral clearance [96]. Indeed, after depletion of Tregs by anti-CD25 antibody in herpes simplex virus (HSV) infected mice, increased CD4^+^ T cell responses, enhanced CD8^+^ proliferative and cytotoxic T cell responses, and increased mucosal antibody levels were reported as compared to nondepleted animals [97, 98]. In addition, viral clearance occurred more rapidly in Treg-depleted mice [99]. In humans with chronic hepatitis B virus (HBV) and HCV infection, an increase in peripheral CD4^+^CD25^+^ Tregs, as compared to healthy individuals, has been described [100, 101]. Moreover, these Tregs are able to suppress HCV-specific CD8^+^ T cell immune responses [102, 103]. Besides increased levels of Tregs in patients, IL-10 producing Tr1 cells could also be isolated and cloned from patients with chronic HCV infection, but not from patients who cleared the infection [104].

Following the demonstration of the role of Tregs in suppressing antiviral immune responses, several in vitro studies showed that depletion of Tregs from peripheral blood of virally infected patients results in increased T cell responses to HBV, HCV, cytomegalovirus (CMV), and human immunodeficiency virus (HIV) [105, 106]. While the presented results are clear for HBV, HCV, and CMV infections, the influence of Tregs during HIV infection might be more complex. The data provided so far do not provide conclusive evidence whether Tregs in HIV-infected individuals limit or contribute to immune activation, which results in immune dysfunction. On the one hand, the frequency of Tregs inversely correlates with the magnitude of SIV/HIV-specific CTL responses [105, 107]. Moreover, patients with long-term nonprogressing disease have low numbers of Tregs in different lymphoid compartments, further supporting the notion that Tregs prevent efficient anti-HIV responses [108]. On the other hand, the number of circulating Tregs has also been reported to be decreased in chronically HIV-infected patients, and this correlates with hyperactivation [109, 110]. This observed decrease of Treg frequency in blood can be due to altered trafficking and/or accumulation of Tregs into lymphoid tissues, and it warrants further investigation [108, 111].

## 5. MANIPULATING REGULATORY T CELLS FOR THERAPEUTIC APPLICATIONS

The observations mentioned above have therapeutic implications for targeting Tregs in human disease. Most advanced studies regarding this topic have been performed in the field of autoimmunity, where the challenge is to enhance Treg responses against those self-antigens involved in disease progression. Moreover, it is clear that experimental strategies to activate and expand self-reactive Tregs in order to diminish tissue damage may also be applicable to control virus-induced immune pathology or to inhibit transplant graft rejection. Alternatively, limiting or preventing Treg responses would be required to enhance insufficient immune responses against certain viral antigens and tumor-associated antigens (TAAs). Below we discuss several possibilities to manipulate Treg function in vitro and in vivo.

### 5.1. Treg-inducing therapies

Nonspecific (experimental) therapies using antibodies and anti-inflammatory cytokines have been documented to modify Treg function. For example, treatment with infliximab (anti-TNF-*α*) in RA was able to restore the defective suppressive function of Tregs and to increase the number of peripheral Tregs [69]. Also, administration of nonmitogenic anti-CD3 monoclonal antibodies [112] and immunomodulatory cytokines, such as TGF-*β* [113], are Treg-modulating strategies currently under investigation. Several in vitro studies have revealed a role for costimulation through CD28 to promote Treg proliferation [114, 115]. In support of this concept, superagonistic anti-CD28 antibodies, which probably cause augmented CD28 signaling, are particularly effective at supporting Treg expansion in vivo [116, 117]. However, it is worth remembering that these antibodies, when tested in six healthy volunteers in a clinical trial, sent all these healthy male subjects to critical care, unlike two additional participants who had received a placebo. What probably happened is that, since CD28 receptors are found on different cells of the immune system, this may have caused mass activation of the immune system, causing a devastating “cytokine storm” [118, 119].

Although only the antigen-nonspecific approaches yielded clinical benefit so far, the dramatic outcome of the clinical trial mentioned above or the risk of opportunistic infections using such antigen-nonspecific strategies might become problematic. Therefore, current research focuses on the development of novel antigen-specific Treg therapies in order to reduce or prevent immune-mediated pathologies by selective enhancement of antigen-specific Treg populations in vitro or in vivo. Recent studies have investigated the potential to isolate CD4^+^CD25^+^ Tregs from peripheral blood and to (antigen-nonspecifically) expand them in vitro for subsequent adoptive transfer in patients, in order to modulate ongoing immune responses in vivo. These expanded cells retained expression of CD25, FOXP3, and lymph node homing receptors. Moreover, such in vitro expanded Tregs appeared to be more efficient in in vitro suppression assays as compared to freshly isolated Tregs [120, 121]. In addition, in vitro expansion protocols for Tregs can be combined with strategies to generate antigen-specific artificial Tregs. In this context, Mekala and Geiger [122] described that genetic modification of polyclonal Tregs with a chimeric receptor consisting of a myelin basic protein (MBP) epitope bound to the extracellular and transmembrane domains of an MHC linked to the cytoplasmic domain of the TCR *ζ*-chain results in functional Treg activation upon recognition of these modified Tregs by MBP-specific T cells. These receptor-modified CD4^+^CD25^+^ Tregs inhibited both the onset and the development of experimental autoimmune encephalomyelitis (EAE). This inhibition only occurred when EAE was induced by MBP, but not by any other known EAE autoantigen. While the strategy described above alters the fundamental mechanism of Treg biology, Jaeckel et al. [123] developed another strategy based on transduction of naive CD4^+^ T cells from nonobese diabetic (NOD) mice with FOXP3, the trancription factor associated with Treg development and function. These FOXP3-transduced CD4^+^ T cells produce IL-10 and they are able to suppress CD4^+^ T cell proliferation. However, a therapeutic effect was only observed when FOXP3 was transduced in T cells from TCR transgenic mice that recognize a pancreatic islet antigen. Again, this indicates that antigen specificity of Tregs will be important for therapeutic efficacy.

Next to genetic modification of T cells, in order to obtain antigen-specific artificial Tregs, the fact that CD4^+^- CD25^+^FOXP3^+^ Tregs can also be generated in the periphery might have important clinical implications as no clear phenotypic or functional differences have been observed that distinguish them from thymic-derived Tregs [21, 124, 125]. In this context, it has been demonstrated in several studies that DCs can induce different subsets of Tregs. Moreover, it has been demonstrated that tolerogenic DCs loaded with specific antigen in combination with IL-2 are able to expand antigen-specific Tregs ex vivo [114, 126–128]. Alternatively, in vivo targeting of DCs in a steady-state condition by anti-DEC-205 antibody preferentially increases the number of CD4^+^CD25^+^ Tregs [129, 130]. Importantly, such strategies require that DCs remain in their tolerogenic state in order to prevent immune activation. The latter is currently a subject of major interest.

### 5.2. Treg-depleting strategies

As mentioned above, limiting or preventing Treg responses might be desired to enhance insufficient immune responses against certain viral and tumor antigens. Elimination of Tregs by CD25^+^ T cell depletion with ONTAK has recently been evaluated in clinical trials. ONTAK or denileukin diftitox is a ligand-toxin fusion protein that consists of full-length IL-2 fused to the translocating and enzymatically active domain of diphtheria toxin [131]. Several studies demonstrated that administration of ONTAK in cancer patients results in reduced prevalence of peripheral Tregs and increased effector T cell activation [132–134]. Moreover, Dannull et al. [132] showed that administration of ONTAK combined with vaccination, with DCs transfected with total tumor RNA, led to improved stimulation of tumor-specific effector T cells as compared to DC vaccination alone.

However, because of their nonexclusive phenotype, depletion of Tregs in vivo is difficult to achieve and may also lead to severe autoimmune complications (“collateral damage” [135]). Therefore, interfering with Treg activity would be a more appropriate strategy. Enhanced immune responses have been observed after addition of anti-GITR antibodies. Ligation of GITR on Tregs results in abrogation of their suppressive function [136]. Moreover, ligation of GITR on effector T cells provides effector T cells with additional costimulation and makes them refractory to the suppressive effects of Tregs [137, 138]. Alternatively, it has been demonstrated that anti-CTLA-4 antibody inhibits the suppressive activity of Tregs in patients with malignant melanoma. Effective reduction in tumor mass was shown in approximately 20% of patients. Interestingly, reduction of tumor size was linked to the development of severe, but manageable, autoimmune syndromes [41–44]. However, in cancer patients treated with the anti-CTLA-4 antibody, no effect was observed on the number or the suppressive activity of peripheral blood Tregs. This indicates that CTLA-4 signaling might represent a regulatory mechanism independent, at least in part, of Tregs [139]. Moreover, it is also demonstrated that both mechanisms, Treg depletion and CTLA-4 blockade, can work synergistically [140] on enhancing antitumor immunity in experimental B16 melanoma.

Finally, another potential strategy to interfere with Treg function is to target molecules involved in Treg trafficking. Blocking CCL22 has been proposed to reduce Treg trafficking in ovarian cancer in order to prevent their inhibitory function on APCs and on tumor-specific T cells [91].

## 6. CONCLUSIONS

Since the reappraisal of suppressor T cells by the pioneering work of Sakaguchi et al. [3], the field of immune control by Tregs has been progressing exponentially. Despite recent advances, several major questions remain regarding their interactions with other cells of the immune system, leading to their suppressive activity. The quest for more specific markers on naturally occurring or induced Tregs will ultimately lead to improved methods to isolate and functionally characterize these Treg subsets. Better insights will then improve the design of new and better immunotherapies that should be able to (i) antigen-specifically enhance immune responses against pathogens and tumors or (ii) antigen-specifically abrogate immune responses against self-antigens or cell and organ transplants.

## Figures and Tables

**Table 1:** Different subsets of regulatory T cells.

Cell type	Phenotype	Suggested immunosuppressive mechanism
CD4^+^ regulatory T cells
Thymic-derived naturally	CD4^+^CD25^+^FOXP3^+^	Cell-cell contact-dependent in vitro (CTLA-4); cell-cell contact- and cytokine-dependent in vivo (IL-10 and TGF-*β*)
occurring Treg		
Peripheral-induced naturally	CD4^+^CD25^+^FOXP3^+^	
occurring Treg		
Tr1 cells	CD4^+^CD25^±^FOXP3^−^IL-10hi	Cell-cell contact
		Cytokine-mediated (IL-10 production)
Th3 cells	CD4^+^CD25^±^FOXP3^−^TGF-*β*hi	Cytokine-mediated (TGF-*β* production)
TGF-*β*/IL-10 double-positive CD4^+^ Treg	TGF-*β*/IL-10 double-positive	Cytokine-mediated (IL-10)
	CD4^+^CD25^−^FOXP3^−^	and (TGF-*β* production)
CD8^+^ regulatory T cells
T suppressor cells (Ts)	CD8^+^CD28^−^	Cell-cell contact-dependent (CTLA-4)
IL-10 producing CD8 T cells	CD8^+^IL-10^+^	Cytokine-mediated (IL-10 production)
